# Analysis of Two SusE-Like Enzymes From *Bacteroides thetaiotaomicron* Reveals a Potential Degradative Capacity for This Protein Family

**DOI:** 10.3389/fmicb.2021.645765

**Published:** 2021-06-04

**Authors:** James Stevenson, Maria Ngo, Alicia Brandt, Joel T. Weadge, Michael D. L. Suits

**Affiliations:** ^1^Department of Chemistry and Biochemistry, Wilfrid Laurier University, Waterloo, ON, Canada; ^2^Groningen Biomolecular Sciences and Biotechnology Institute, Faculty of Science and Engineering, University of Groningen, Groningen, Netherlands; ^3^Department of Biology, Wilfrid Laurier University, Waterloo, ON, Canada

**Keywords:** *Bacteroides thetaiotaomicron*, galactose, starch utilization system, crystallography, SAXS, SusE, SusE-like

## Abstract

*Bacteroides thetaiotaomicron* is a major constituent of the human gut microbiome and recognized as a prolific degrader of diverse and complex carbohydrates. This capacity is due to the large number of glycan-depolymerization and acquisition systems that are encoded by gene clusters known as polysaccharide utilization loci (PUL), with the starch utilization system (Sus) serving as the established model. Sharing features with the Sus are Sus-like systems, that require the presence of a specific membrane transporter and surface lipoprotein to be classified as Sus-like. Sus-like import loci are extremely varied with respect to any additional protein components encoded, that would effectively modify the functionality of the degradative and import action of each locus. Herein we have identified eight Sus-like systems in *B. thetaiotaomicron* that share the feature of a homologous SusE-like factor encoded immediately downstream from the transporter/lipoprotein duo *sus*C/D. Two SusE-like proteins from these systems, BT2857 and BT3158, were characterized by X-ray crystallography and BT2857 was further analyzed by small-angle X-ray scattering. The SusE-like proteins were found to be composed of a conserved three domain architecture: a partially disordered N-terminal domain that is predicted to be proximal to the membrane and structurally homologous to an FN3-like bundle, a middle β-sandwich domain, and a C-terminal domain homologous to family 32 carbohydrate-binding modules, that bind to galactose. Structural comparisons of SusE with SusE-like proteins suggested only a small structural divergence has occurred. However, functional analyses with BT2857 and BT3158 revealed that the SusE-like proteins exhibited galactosidase activity with para-nitrophenyl-β-D-galactopyranoside and α-(1,4)-lactose substrates, that has not been demonstrated for SusE proteins. Using a series of domain truncations of BT2857, the predominant β-D-galactosidase activity is suggested to be localized to the C-terminal DUF5126 domain that would be most distal from the outer membrane. The expanded functionality we have observed with these SusE-like proteins provides a plausible explanation of how Sus-like systems are adapted to target more diverse groups of carbohydrates, when compared to their Sus counterparts.

## Introduction

The microbial composition of the human gut microbiome has important implications for both the health of the intestine and the body as a whole (Bäckhed et al., 2005; Sonnenburg and Bäckhed, 2016; Hall et al., 2017). In addition to the maintenance of digestive and physiological health, the gut microbiota contributes to digestive efficiency by making otherwise inaccessible caloric sources available (Turnbaugh et al., 2006; Larsson et al., 2012). Bacterial fermentation of undigested soluble carbohydrates produces short-chain fatty acids that are readily absorbed by the host (Russell et al., 2013); contributing up to 10% of daily caloric intake depending on the particular makeup of host diet and microbiome (Hooper et al., 2002). In addition to undigested dietary carbohydrates, host-secreted polysaccharides, like the chondroitin and hyaluronic acid glycosaminoglycans (Ndeh et al., 2018), are also a prominent nutrient source for intestinal microbes (Martens et al., 2009a). The abundance and diversity of host and dietary carbohydrates, combined with factors such as the high moisture content and warm temperature, creates an ideal ecological niche within the body for the establishment of a large and diverse microbiome (Almeida et al., 2019). As such, several prominent members of the human gut microbiota have evolved complex pathways that allow for the degradation of diverse carbohydrate sources (Kaoutari et al., 2013).

*Bacteroides thetaiotaomicron* is a major constituent of the human gut microbiome and recognized as a prolific degrader of diverse and complex carbohydrates, which is why it is often used to discover new carbohydrate-active enzymes (CAZymes) and related factors. In *B. thetaiotaomicron*, carbohydrate-processing pathways are tightly regulated by gene clusters known as polysaccharide utilization loci (PUL) (Sonnenburg et al., 2010; Ravcheev et al., 2013; Huang et al., 2018). These PULs can cover up to 18% of the genome of *B. thetaiotaomicron* and may constitute 866 distinct genes for carbohydrate processing (Martens et al., 2009a). Transcription of a PUL is activated by the presence of the particular carbohydrate substrate targeted by the protein products of these genes (Martens et al., 2009b, 2011), with transcriptional regulation occurring through a variety of mechanisms (Cho et al., 2001; Ravcheev et al., 2013). The starch utilization system (Sus) has emerged as a model system for the degradation and import of carbohydrates from the extracellular environment into bacterial cells, with the prototypical PUL coding for a total of eight proteins (Foley et al., 2016). This particular model of carbohydrate acquisition has subsequently been used to identify several other PUL-encoded systems as Sus-like (TerAvest et al., 2014), wherein each Sus-like locus minimally contains homologs to the TonB dependent porin, SusC, and the outer-membrane glycan-binding protein, SusD (Bolam and Koropatkin, 2012). A number of Sus-like PULs code for products homologous to classic Sus proteins other than SusC/SusD, though the current research on these homologs remains sparse despite their prevalence in the genomes of various PUL-coding organisms, including *B. thetaiotaomicron* (Martens et al., 2009a). One such group of homologous proteins are often found to be positionally conserved within certain PULs immediately downstream from the SusC-/SusD encoding gene pair, which has led to their designation as “SusE-like” proteins.

In the prototypical Sus PUL found in *B. thetaiotaomicron* (e.g., BT3700), the proximal downstream gene from *susC*/*susD* encodes for SusE, which is a protein responsible for binding and orienting α-glucan at the cell surface for transport through SusC (Foley et al., 2016). Previous research has also shown that this SusE (along with SusF) forms the center around which the membrane component of Sus complexes assemble (Foley et al., 2016; Tuson et al., 2018). Major structural features of SusE proteins include (i) an N-terminal cysteine immediately following each signal peptide region linking the protein to the membrane, (ii) a linker region with an Ig-like or FN3 fold (referred to as E_a_), and (iii) two β-sandwich fold binding domains (E_b_ and E_c_). Despite the prevalence of SusE-like proteins in *B. thetaiotaomicron* PULs, only one has so far has been characterized structurally and functionally; a six-domain glycan-binding protein involved in the import and degradation of the glycosaminoglycan heparin-/heparin-sulfate (Cartmell et al., 2017). While this system represents a significant deviation in size and structure compared to the prototypical three-domain SusE, it underscores the ability of this family of proteins to bind a diversity of polysaccharides.

To gain a greater understanding of the differences in breakdown and transport in Sus and Sus-like systems of *B. thetaiotaomicron*, we identified a series of PULs that contained SusE-like proteins and initiated the structural and functional characterization of these factors. Structural comparison of SusE-like proteins with SusE from BT3700, indicated that SusE-like proteins contain shorter sections of secondary structure elements, while retaining a global fold similar to that of the prototypical SusE and carbohydrate-binding module (CBM) family 32. Surprisingly, these SusE-like proteins also possess the ability to degrade certain galactose-containing oligosaccharides, while their SusE counterparts have only been shown to bind and position carbohydrates for transport via the SusC porin to date.

## Materials and Methods

### Enzyme Production and Purification

#### Bioinformatics Analyses

SusE-like proteins within the *B. thetaiotaomicron* ATCC 29148 genome were initially identified via a DELTA-BLAST search using BT2857 (Boratyn et al., 2012). The encoded amino acid sequences were compared to the prototypical SusE using EMBOSS Matcher and ClustalW. Domains of interest in the amino acid sequences of target proteins were suggested using BLASTp (Altschul et al., 1997). Additional members in the associated SusE-like PULs were recovered via the Kyoto Encyclopedia of Genes and Genomes (KEGG) (Kanehisa and Goto, 2000). Predicted physiochemical properties of target and truncated target proteins were assessed using the ExPASy ProtParam online tool (Gasteiger et al., 2005).

#### Cloning and Transformation

BT2857 and BT3158 genes were amplified from *B. thetaiotaomicron* (ATCC 29148) genomic DNA by polymerase chain reaction (PCR) using primers containing restriction sites *Nde*I and *Xho*I (Supplementary Table 1). Standard PCR conditions for 35 cycles was performed with Pfu DNA polymerase, and the ∼1.3 kb amplicons were subsequently digested with *Nde*I and *Xho*I before being ligated into complimentary digested pET15b plasmid treated with alkaline phosphatase via ligation-independent cloning (Aslanidis and de Jong, 1990). Two truncations of BT2857; one spanning N-terminal region (DUF4959/5126) and another the C-terminal region (DUF5000), were codon optimized synthetic genes cloned into the pET21b expression vector (Bio Basic Inc.), referred herein as BT2857N and BT2857C, respectively. Plasmids were transformed into calcium-competent *Escherichia coli* cells [NEB 5-alpha, BL21 (DE3), Tuner (DE3)] via heat shock and plated on LB Miller agar supplemented with 100 μg/ml ampicillin. Similar protocols were used to clone and express other SusE-like factors BT2109, BT2918, and BT2966. We were unsuccessful in cloning BT2903.

### Growth Conditions for *E. coli* Expression of Recombinant Proteins

Transformed BL21 (DE3) and Tuner (DE3) cells were cultured overnight in 10 mL of LB media before being inoculated into flasks containing 1 L LB media supplemented with 100 μg/ml ampicillin. Flasks were incubated at 37°C and shaken at 240 rpm until cultures reached an optical density (O.D.) of 0.6–0.8 at 600 nm, at which time isopropyl-β-D-1-thiogalactopyranoside (IPTG) was added to a final concentration of 1 mM to induce recombinant protein expression. Culture conditions were subsequently reduced to 16°C and 160 rpm, and incubated for 18 h. 10 mL overnight cultures used to inoculate 1 L of minimal media containing selenomethionine (Molecular Dimensions Ltd.) (Niedzialkowska et al., 2016) were pelleted via centrifugation and resuspended in sterile water twice before being added to media for protein production. These 1 L cultures were then incubated in the same manner as LB media cultures to an O.D._600__nm_ of 0.6–0.8 before having expression initiated with IPTG and being reduced to 16°C/160 rpm overnight. In all cases, cells were harvested via centrifugation at 4800 × *g* for 8 min at 4°C. BT2918 did not readily express and purify.

### Cell Lysis and Protein Purification

Bacterial cells were lysed by the osmotic shock method. Cells were initially resuspension in chilled 50 mM Tris:HCl (pH 8.0) buffer, 730 mM sucrose, and 1 mg/mL lysozyme. Homogenized suspensions were subsequently treated with the addition of chilled sodium deoxycholate/Triton X-100 solution to final concentrations of 1 and 2% (w/v), respectively, and incubated for 10 min. Suspensions were further liquefied through treatment with DNase I at 2 μg/ml for 5 min. Cellular debris was captured by centrifugation at 30,000 × *g* for 45 min at 4°C and the supernatants were retained. Initial purification of SusE-like protein was performed via immobilized-metal affinity chromatography (IMAC). All solutions used throughout lysis and IMAC purification were buffered using 50 mM of Tris:HCl (pH 8.0) supplemented with 300 mM NaCl. Clarified cell extracts were loaded through equilibrated Ni-NTA resin, followed by 15 column volumes of wash with buffer supplemented with 5 mM imidazole solution, and proteins eluted over an increasing 5–250 mM linear gradient of imidazole. Proteins expressed in Tuner (DE3) were purified using brand new resin. Samples were dialyzed first against Milli-Q dH_2_O then 50 mM Tris:HCl (pH 8.0) buffer overnight (4°C). Secondary purification of proteins was performed via anion-exchange chromatography (GE Healthcare 5 ml HiTrap Q FF column on an ÄKTA pure FPLC purification system) using a 0–1 M NaCl gradient to fractionally elute proteins. The purity of each fraction was assessed through SDS-PAGE analysis and pure samples were pooled before concentration via ultrafiltration (Amicon Ultra-15, 10,000 Da molecular weight cut-off). Protein concentration was determined in sample solutions via absorbance spectroscopy using the determined extinction coefficients of 74175, 19370, 48930, and 94770 M^–1^ cm^–1^ for BT2857, BT2857N, BT2857C, and BT3158, respectively.

### Crystallization and X-Ray Diffraction Analysis

Crystals of BT2857C and BT3158 were obtained using the hanging-drop vapor diffusion method at 18°C, with one molecule and two molecules in the asymmetric units, respectively. While we were able to successfully express and purify BT2109, BT2857, and BT2966, we focused our resources on BT2857 and BT3158 as crystallization proceeded more readily with these two. Extensive crystallization attempts with full-length BT2857 or the BT2857N failed to yield diffraction-quality crystals. However, BT2857C readily crystallized in plate-like morphology against reservoir solution comprised by 0.1 M sodium acetate buffer at pH 4.5, 6.0% (w/v) PEG 3.35K, 5% (v/v) ethylene glycol, 0.2 M ammonium acetate, with droplets containing 3 μl of protein solution at 25 mg/ml and 3 μl reservoir solution. BT2857C was also crystallized as a selenomethionine derivative in similar conditions. BT3158 crystals developed against a reservoir solution containing 24% (w/v) PEG4000, 8.0% (v/v) ethylene glycol, 0.4 M MgCl_2_, and 100 mM Tris:HCl (pH 8.5) buffer. BT3158 crystallization droplets contained 3 μl protein at 20 mg/ml (50 mM Tris:HCl (pH 8.0), 300 mM NaCl and 5 mM DTT) and 1.5 μl reservoir solution, and have an irregular rectangular prism-like morphology. In both cases, crystals selected for diffraction analysis were prepared by briefly soaking crystals in cryoprotectant solutions consisting of the reservoir conditions of the respective crystals supplemented with 30% (v/v) ethylene glycol and snap frozen in liquid nitrogen. X-ray diffraction data were collected at the Canadian Light Source (Saskatoon, SK, Canada) using 0.2° oscillations per image, covering 360° total. Data were indexed and processed using the on-site Autoprocess pipeline (Fodje et al., 2012) and SCALA (Evans, 2011), and the initial substructure of selenium atom positions were identified and refined using SHELX (Usón and Sheldrick, 2018). Initial model building was performed with PHENIX Autobuild (Terwilliger et al., 2007), that were iteratively built and refined with COOT (Winn et al., 2011). Models were visualized using UCSF Chimera (Pettersen et al., 2004). Data processing and structure refinement statistics for BT2857C and BT3158 are presented in Table 1.

**TABLE 1:** Diffraction data collection and structure refinement statistics.

	**BT2857C**	**BT3158**
PDBID	7M1A	7M1B*
Resolution (Å)	60.50–1.42 (1.50–1.42)	40.50–1.50 (1.58–1.50)
Space Group	P2_1_	C2_1_
**Cell Dimensions**		
*a*, *b*, *c* (Å)	59.30, 61.08, 61.76	74.65, 48.79, 112.66
α, β, γ (°)	90.00, 101.59, 90.00	90.00, 103.60, 90.00
Measured Reflections	517327	388958
Unique Reflections	81091	56259
Multiplicity	6.4 (5.4)	6.1 (6.1)
Completeness (%)	99.5 (97.5)	99.9 (99.9)
R_measure_	0.080 (0.285)	0.053 (0.483)
I/σ(I)	15.6 (5.6)	18.3 (3.6)
CC_1__/__2_ (%)	0.999 (0.978)	0.999 (0.939)
**Structure Refinement**		
Resolution (Å)	1.42	1.50
*R*_work_/*R*_free_	0.167/0.179	0.201/0.237
**No. of atoms**		
Protein	2878	2164
Calcium	2	N/A
Solvent	403	229
***B***-**factors**		
Protein Chains	11.96	28.03
Calcium	8.62	N/A
Solvent	23.89	37.09
**RMSD**		
Bond lengths (Å)	0.017	0.017
Bond angles (°)	1.338	1.337
**Ramachandran**		
Preferred (%)	331 (94.6)	250 (95.4)
Allowed (%)	18 (5.1)	12 (4.6)
Disallowed (%)	1 (0.3)	0 (0)

*Due to the absence of continuous electron density, the deposited coordinates are lacking the N-terminal domain.

### Small-Angle X-ray Scattering

To prepare BT2857 for small-angle X-ray scattering (SAXS) analysis, an unconcentrated sample of protein was dialyzed against 50 mM Tris:HCl (pH 8.0) at 4°C overnight. Samples were then centrifuged, supernatants collected, and concentrated to 20 mg/ml. This concentrate was diluted with filtered dialysis buffer to 2, 5, and 10 mg/ml. 30 μl aliquots of protein solutions and duplicate of the dialysis buffer were pipetted into an Axygen 96-well full-skirt PCR microplate and the plate was sealed. Microplate was then delivered on ice to the Advanced Light Source at the Lawrence Berkeley National Laboratory (Berkeley, CA, United States) for data collection on the SIBYLS beamline. An exposure time of 0.3 s per image was used with 34 images collected per sample. The wavelength used was 1.0 Å with a flux of 1013 photons/s and a sample-to-detector distance of 1500 mm. All sampling was conducted at 10°C. Data were analyzed and a model was fitted using the software ScÅtter^1^ and the ATSAS suite of programs (Franke et al., 2017). Optimal fitting of the pair distance distribution function was found at a d_max_ of 219.5 Å and DAMMIF was used to conduct 20 rounds of *ab initio* modeling (assuming P1 symmetry and unknown anisometry) followed by DAMAVER averaging. Analysis with P2 symmetry were associated with elevated statistical indices. Low density regions were removed using DAMFILT.

### Functional Assay

Target and truncated proteins were first tested for potential activity against saccharides containing galactose via colorimetric assays utilizing the various substrate analogs including para-nitrophenyl-α-/para-nitrophenyl-β-D-galactopyranoside (*p*NP-α-Gal and *p*NP-β-Gal). As bioinformatics analysis suggested homology with galactose-binding proteins, we started with *p*NP-α-Gal and *p*NP-β-Gal as bioinformatics analysis suggested that the C-terminal domain of the SusE-like proteins shared homology to galactose binding domain proteins. Additionally, we assayed other *p*NP-reporters available to us including *o*NP-β-glucose, *p*NP-α-glucose, *p*NP-α-mannose, *p*NP-α-rhamnopyranoside, and *p*NP-β-glucuronide. The pH optima for each protein were determined with these analogs in 100 mM McIlvaine or phosphate-buffered solutions from pH 5.8–9.2. Michaelis–Menten kinetic analysis was then conducted using the same substrate analogs at room temperature in buffering conditions found to be optimal. Mixtures contained a combination of 100 mM phosphate-based buffer and 100 mM buffer + 10 mM *p*NP-analog, with solutions totaling 248 μl and concentrations of *p*NP-analog ranging from 0, 0.01 to 6 mM, with the reactions being initiated by the addition of 2 μl of 625 μM protein sample. The amounts of para-nitrophenolate liberated from *p*NP-α/β-Gal were measured at 405_nm_ (BioTek Cytation5). Reactions were conducted in triplicate and data was analyzed to a non-linear model using Prism. The possibility of endogenous BL21:DE3 β-galactosidase causing a false positive in *p*NP release was ruled out by testing of protein expressed in lacZY-deficient Tuner (DE3) *E. coli* cells purified using brand new resin. A series of buffered blanks containing substrate analogs were analyzed alongside wells containing protein in order to correct for auto-hydrolysis of para-nitrophenyl group.

Following kinetic analysis, targets were assessed for degradative capability against a variety of natural carbohydrate substrates containing galactose or galactose-like monomers via thin-layer chromatography (TLC). 5 mg of each selected carbohydrate substrate was dissolved in 495 μl of Milli-Q dH_2_O and reactions were initiated with addition of 5 μl of protein solution concentrated to 25 mg/ml before being incubated at 37°C 240 rpm for 18 h. 500 μl reagent ethanol was added to reactions and allowed to sit for 1 h at room temperature to arrest reactions, after which tubes were spun at 14,000 × g for 30 min to pellet denatured protein. Supernatant was pipetted out of tubes and solutions were concentrated via centrifugal evaporation at 40°C until a volume of 50–100 μl had been reached. Solutions were pipetted on to silica gel TLC plates (Millipore Sigma) developed using a mobile phase solvent containing a 2:1:1 ratio of 1-butanol, glacial acetic acid, and Milli-Q dH_2_O. After development TLC plates were marked, dried, and visualized through the application of a 1% (w/v) p-anisidine solution (10% (v/v) methanol, 90% (v/v) 1-butanol solvent) and heating of the plate at 110°C for 10 min. Ruling out residual BL21:DE3 activity was again done by conducting digestions with protein expressed in Tuner (DE3) cells.

## Results and Discussion

### Architecture of *B. thetaiotaomicron* PULs Containing SusE-Like Genes

**FIGURE 1:**
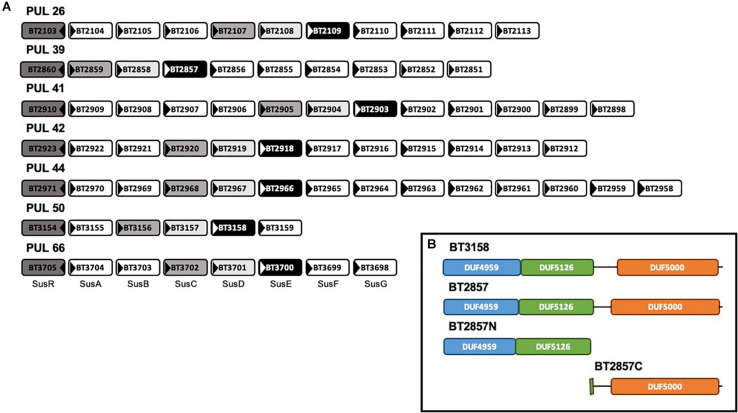
Genomic organization of six SusE-like *B. thetaiotaomicron* polysaccharide utilization loci and the model Sus polysaccharide utilization loci (PUL66) and domain architecture of SusE-like target BT2857. **(A)** Located at the beginning of each Sus and Sus-like loci is the gene encoding a regulatory protein, illustrated by each dark gray box. Medium gray and light gray boxes indicate SusC- and SusD(-like) proteins, respectively. Black boxes indicate homologous SusE-like proteins. White boxes represent miscellaneous members of each PUL (Martens et al., 2009a). This architecture organization highlighted PULs 26, 39, 41, 42, 44, and 50 to have these unique features within the *B. thetaiotaomicron* ATCC 29148 genome. To our knowledge, none of these PULs have had any activity attributed. **(B)** Inset–outer membrane-targeting signal peptides covering the first 19–20 amino acids were truncated from the expressed constructs. The first amino acid in each DUF4959 was found to be cysteine suggesting that SusE-like proteins, like their model counterpart, are anchored to the membrane via an attached lipid (Tuson et al., 2018).

The six Sus-like PULs identified in this study demonstrated the requisite *sus*C/*sus*D synteny and were selected for further study due to the recurring presence of *sus*E-like genes with high similarity surrounding the *sus*C/*sus*D duo. The prototypical Sus PUL (Figure 1; PUL 66) contains a total of eight genes dedicated to the degradation of starch; a relatively simple polysaccharide that is a homopolymer with two types of alternating glycosidic bonds (Nelson and Cox, 2008). Many of these are dedicated to regulation, carbohydrate recognition and uptake. As a contrast, the *B. thetaiotaomicron* PULs containing Sus-like operons were found to be substantially varied in both the number and putative functions of the encoded gene products when compared to the model Sus system. The composition of the Sus-like operons varies between six and 14 genes, with BT2857 and BT3158 that were selected for further study belonging to ten- and six-membered loci, respectively. The number of genes suggested to be potentially catalytically active in these Sus-like PULs also varied across those identified, with operons predicted to contain as few as one or as many as seven putative catalytic factors, compared to the three present in the prototypical Sus PUL (Figure 1). This potential enzymatic diversity could suggest that Sus-like operons are able to degrade and import complex carbohydrates. However, to our knowledge, none of these PULs have had any activity or cognate substrates attributed.

SusE and SusE-like proteins are analogous in that they are comprised by a three, mainly β-domain architecture (Figure 1; inset box). To be consistent with the nomenclature used for SusE BT3700, E_a_, E_b_, and E_c_, are analogous to DUF4959, DUF5126, DUF5000 of SusE-like proteins, respectively. This conserved three-domain SusE organization, combined with the positioning within each respective operon, led to our initial classification of this group of genes as SusE-like. Sequence maps of BT3158 and BT2857 (and truncated derivatives thereof used in this study) showing the coverage of each domain can be seen in the inset box of Figure 1.

### Crystal Structures

**FIGURE 2:**
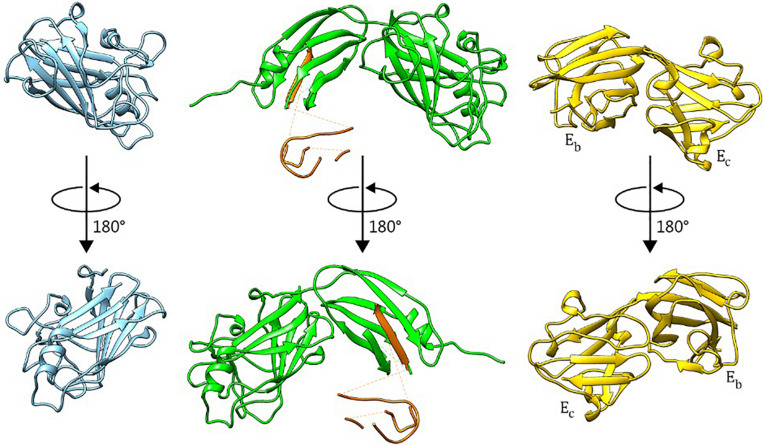
Crystal Structures of BT2857C, BT3158, and BT3700. BT2857C is depicted in blue, BT3158 in green, and BT3700 (RCSB: 4FCH) in yellow. Polyalanine residues used to model sections of the backbone in DUF4959 and DUF5126 of BT3158 are colored orange and left for reference with regards to the positioning of this region.

**FIGURE 3:**
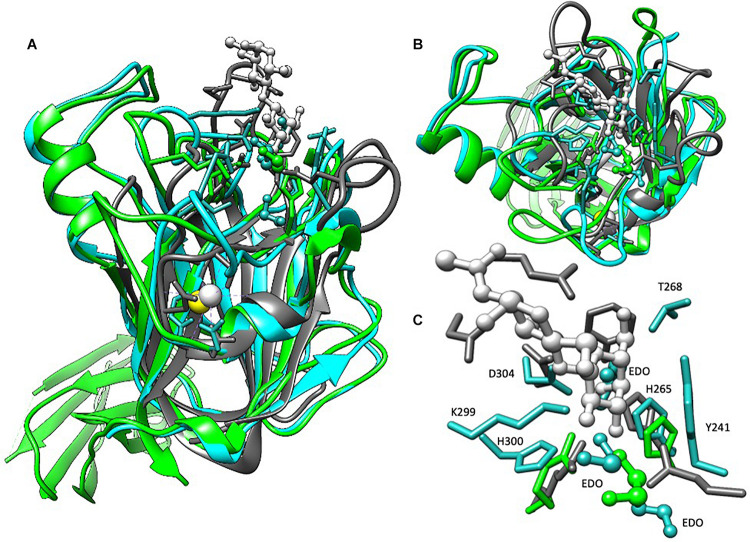
Structural alignment of BT2857C, BT3158, and CBM32 from *C. perfringens*. BT2857C is depicted in blue, BT3158 in green, CBM32 (RCSB: 2J1E) in gray, and associated ligands or solvent in ball-and-stick. CBM32 is shown coordinated to β-d-galactosyl-1,4-β-d-N-acetylglucosamine (white). **(A)** There is high structural similarity in the C-terminal domains (DUF5000 or E_c_ domain) of BT2857 and BT3158 with CBM32 from *C. perfringens* (RMSD = 2.051 and 1.991 Å, respectively). The majority of the overall fold is conserved across the three structures but diverge at the carbohydrate accommodation portion of CBM32. **(B)** Intriguingly, in both BT2857C and BT3158 crystallographic ethylene glycols (EDO) that was supplemented during crystallization and used as a cryoprotectant were found in the carbohydrate accommodation position of CBM32. **(C)** Residues contributed (numbering presented for BT2857) at the CBM accommodation site diverge across the three proteins, apart from a conserved histidine, H265 which is presented by all three proteins.

The BT2857C construct crystallized in the P2_1_ space group with two molecules in the asymmetric unit, and high-quality diffraction data was processed to 1.42 Å resolution (Table 1). The structure of BT2857C adopts a β-sandwich fold with extensive loop regions and high similarity to the equivalent domain of BT3158 (Figures 2, 3A). BT3158 crystallized in a C2_1_ space group, with one molecule in the asymmetric unit. Diffraction data for BT3158 were processed to a maximum resolution of 1.50 Å (Table 1). Despite performing diffraction analyses on multiple crystals and no detectable protein degradation observed via SDS-PAGE, discontinuous electron density was characteristic across the majority of the N-terminal DUF4959 domain. Iterative rebuilding and refinement of this region suggested that it is composed mainly of β-strands, consistent with sequence-based homology modeling as an FN3-like bundle domain. This deficiency in electron density across the N-terminal region and sufficient size of the asymmetric unit to accommodate the domains was similar to results seen in the prototypical SusE (Cameron et al., 2012) and E_a_ domain of BT3700 (Foley et al., 2016); thereby suggesting this region is consistently disordered and likely forms a discrete domain that would reside close to the membrane and have flexibility independent of the rest of the protein. This domain also contains a conserved cysteine residue, which when mutated in BT3700 abolished adherence of the protein to the membrane, consistent with this being a lipidation site for SusE-like proteins as well.

Beyond the membrane-proximal DUF4959 partially disordered domain, BT3158 presented a distinct two-domain structure, with backbone residues confidently modeled with the exception of an extended loop (166–173). Residues 128–220 (DUF5126 or E_b_ domain) form a β-barrel structure that shares structural similarity [2.30 Å root-mean-square deviation (RMSD)] with a S7 monobody (PDBID 5NKQ). Structural comparison of this middle domain with the SusE domain Eb suggested some similarity in that only 47 amino acids across the residue region 128–220 of BT3158 aligned (2.24 Å RMSD, PDBID 4FEM) (Cameron et al., 2012).

Structures of the DUF5000 or E_c_ domains (BT2857C and BT3158^*E*223–P440^) were compared via the MatchMaker in Chimera using the Smith-Waterman algorithm, yielding a RMSD of 1.225 Å across C-alpha atoms, and a quality score of 0.768. DUF5000 was also found to share structural and some sequence homology with a family 32 CBM from *Clostridium perfringens*, that interacts with galactose (1.991 Å RMSD, PDBID 2J1E) (Ficko-Blean and Boraston, 2006). There is also high structural similarity of CBM32 with BT2857 (2.015 Å RMSD), including a calcium binding site wherein the calcium is coordinated by the side chains of N250, E391, main chain carbonyl oxygens of Y247, R252, T390, and a water (BT2857 numbering, Figure 3B). This calcium coordination site was absent in the resolved structures of BT3158. The central protein fold for this domain is conserved across BT2857, BT3158, and CBM32, but deviates substantially in the carbohydrate accommodation cleft characterized for CBM32 (Figure 3B). While extensive crystal soaking at low and high pH values with galactose, glucose, α-lactose and IPTG was attempted, these failed to result in any crystallographic complexes that would have aided in further identification of important residues and orientation of domains across different proteins relative to each other. Intriguingly, crystallographic ethylene glycol (EDO) that was supplemented during crystallization and used as a cryoprotectant was found in an equivalent location in BT2857 and BT3158. However, there is a lack of residue conservation at this site with the exception of a conserved histidine, H265 (BT2857 and BT3158). These structural differences illustrate that the SusE-like proteins have deviated from the prototypical SusE and CBM32, particularly in the region responsible for carbohydrate accommodation helices (Figure 3C).

### SAXS-Derived Structure of BT2857

**FIGURE 4:**
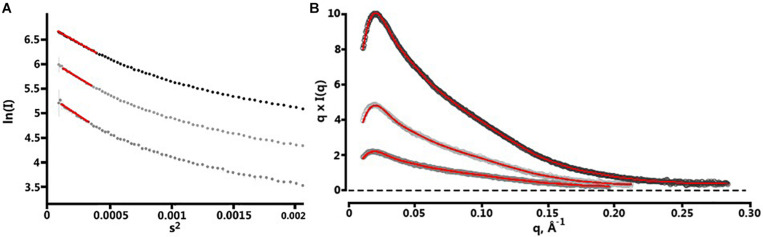
Fitting of SAXS data. **(A)** Plotted data of the Guinier regions (points 1–74) for each concentration of BT2857. 10 mg/ml at top, 5 mg/ml middle, and 2 mg/ml bottom. The linear region is illustrated by red trendlines overlaid on each set of points. 10 mg/ml sample showed linearity from points 4 to 21, 5 mg/ml from 4 to 20, and 2 mg/ml from 4 to 18. **(B)** Depicts the P(r) curves fitted using SPI; concentrations are arranged in the same manner as panel **(A)**.

**FIGURE 5:**
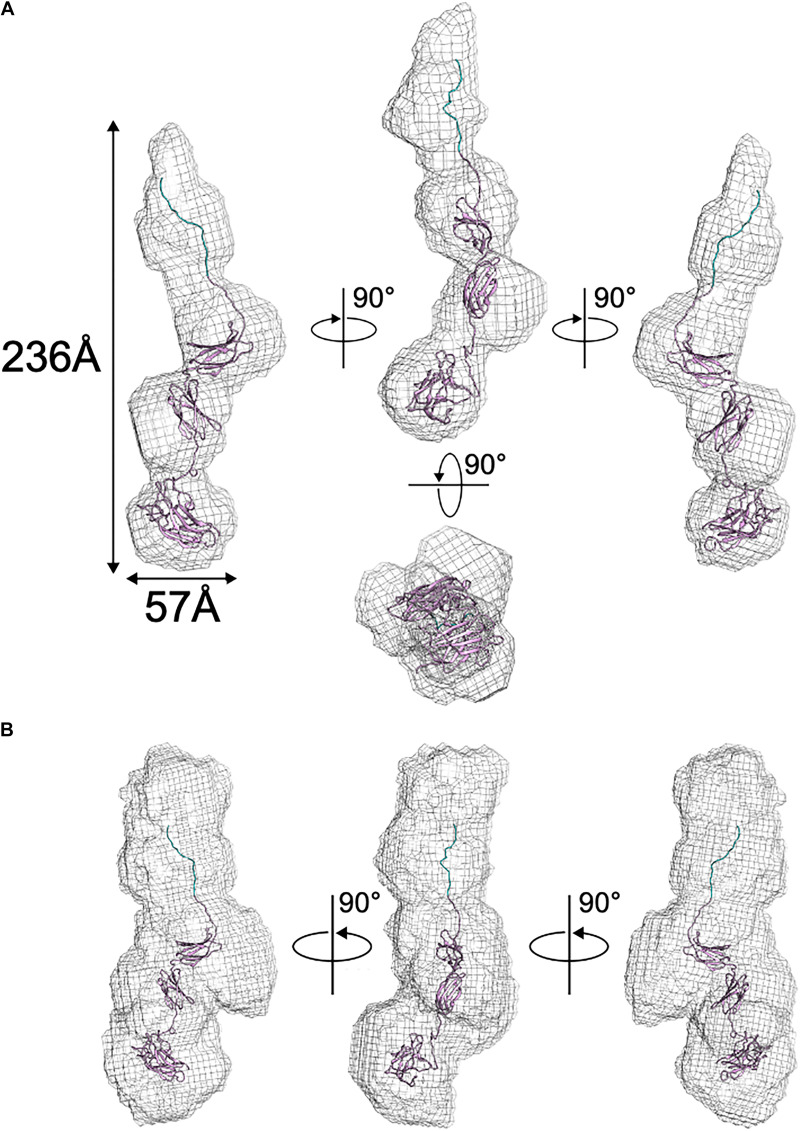
Solution structure of BT2857 as determined by DAMAVER. **(A)** *Ab initio* resolved protein envelope (shown as gray mesh) with fitted BT2857 homology model informed by the structure of BT3158 (shown in ribbons). **(B)** BT2857 contains an N-terminal histidine-tag region (section of ribbon colored blue).

Following the collection of SAXS data, normalization for beam intensity was conducted to generate a one-dimensional scattering profile, which was then buffer subtracted to generate sample profiles. Profiles were then sequentially averaged using the online tool FrameSlice^2^ to compensate for radiation damage in the sample. Of the series of protein concentrations analyzed, 10 mg/mL aliquots were free of unspecific aggregation, so this concentration was used for biological SAXS shape reconstruction and modeling. Kratky analysis was used to assess the extent of protein folding in solution, which displayed a peak at low *q* values followed by a significant plateau at lower *q*^2^ × I(*q*) values, before again rising in the high *q* range. This indicates that BT2857 is mostly folded but has some limited flexibility in solution, which supports our conclusion that multiple orientations and flexibility led to the lack of electron density in DUF4959 noted in the crystal structures. Guinier analysis was used to measure the radius of gyration (*R*_*g*_) as 65.5 Å and cross-sectional radius (*R*_*c*_) as 14.5 Å. Subsequent Porod particle characterization determined a volume parameter (V_p_) of 203970 Å^3^ at a q_max_ of 0.1232, with an exponent of 3.8 and invariant of 0.085. Visualized scattering data can be seen in Figure 4 and processed data in Table 2. DAMSEL output (using SUPCOMB) using the 10 mg/ml sample and the DAMMIF run using the best P(r) fit file resulted in a mean NSD value of 1.029, which seems to be in-line with what was expected based on the crystal structure of BT3158 and BT3700. The lowest DAMMIF NSD found was 0.870 for the 9th model iteration; a model which does not look significantly different from the damfilt model used. The envelope shown in Figure 5 is the one generated by damfilt, using a 10 mg/ml dataset, with the P(r) fittings listed. The chi^2^ value for this model was 0.149. Notably, particle dimensions derived were approximately four times larger than expected for the protein, with SAXSMoW^3^ determining the approximate molecular weight to be ∼193 kDa, while the histidine-tagged construct is predicted to only have a molecular weight of 47.7 kDa. This was the case across data sets for each concentration and buffer subtraction, despite the lack of apparent aggregation and DLS data indicating the protein exists as a monomer in solution. Decreasing *R*_*g*_ approximations and increasing d_max_ values for increasing concentrations suggest interparticle repulsion has likely contributed to the envelopes derived. The final envelope can be seen in Figure 5, fit with a homology model of BT2857 generated by the RaptorX server^4^ using BT3158 as a homology model. The overall structure of the BT2857 molecular envelope is generally cylindrical and 236 Å in length, with the radius of the spherical bulges corresponding to the three DUFs being ∼57 Å in diameter. The fourth domain showing slightly pointed geometry is likely to contain the histidine-tagged region and as such the length of the protein without this section would be closer to 155 Å, assuming the membrane linker extends significantly beyond the DUF4959 domain.

**TABLE 2:** BT2857 SAX data analysis and model refinement.

	**BT2857 Concentration**		
	**10 mg/ml**	**5 mg/ml**	**2 mg/ml**
**Guinier Analysis**			
*I*(0) (cm^–1^)	0.0873	0.0439	0.0210
*R*_g_ (Å)	65.47	66.88	70.87
*q*_min_ (Å^–1^)	0.011	0.011	0.011
*qR*_g_ max (*q*_min_ = 0.011Å^–1^)	1.3	1.3	1.3
***P*(*r*) Analysis**			
*I*(0) (cm^–1^)	0.0801	0.0388	0.0179
*R*_g_ (Å)	64.34	66.74	69.31
*d*_max_ (Å)	219.5	219.5	209.5
*q* range (Å^–1^)	0.011–0.284	0.011–0.212	0.011–0.195
χ^2^ (from SPI)	2.705	2.716	2.454
Porod volume (Å^–3^) (ratio *V*_P_/calculated *M*)	206072	225958	243852

### Kinetics and Degradative Capacity of Protein Targets

**FIGURE 6:**
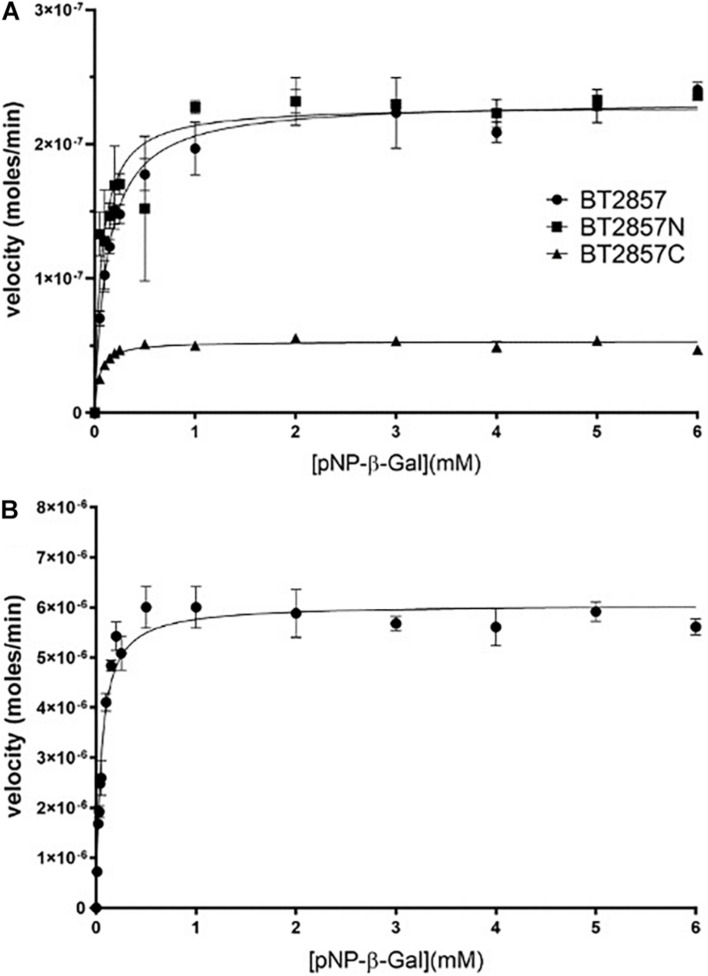
Kinetic analysis of BT2857, BT2857 C-/N-terminal truncations **(A)**, and BT3158 **(B)** using para-nitrophenyl-β-D-galactopyranoside. Reaction volumes of 250 μL were used to assay each protein present at a final concentration of 5 μM (*n* = 3). BT2857 was determined to have a *V*_max_, *K*_M_, and *k*_cat_ of 2.32 × 10^–7^ moles/min (±3.83 × 10^–9^), 0.13 mM (±0.010), and 0.046 s^–1^, respectively. BT2857N was determined to have values of 2.29 × 10^–7^ moles/min (±6.59 × 10^–9^), 0.07 mM (±0.012), and 0.046 s^–1^; while BT2857C generated values of 5.31 × 10^–8^ moles/min (±8.09 × 10^–10^), 0.047 mM (±0.005), and 0.011 s^–1^. BT3158 was determined to have a *V*_max_, *K*_M_, and *k*_cat_ of 6.06 × 10^–6^ moles/min (±1.7 × 10^–7^), 0.052 mM (±0.007), and 1.21 s^–1^, respectively.

*p*NP-α and β-Gal were explored as substrate analogs because the DUF5000 C-terminal region of the protein targets are annotated as galactose-binding domain-like. Both BT3158, as well as BT2857 (and truncations thereof) demonstrated activity against *p*NP-β-Gal, but not *p*NP-α-Gal. Using *p*NP-β-Gal, a pH optima profile for both constructs was performed, with the highest activity for full-length BT2857 (and truncations thereof) determined to be 8.0, with a pH activity range of pH 5.8–9.2. In contrast, the pH optimum for BT3158 was slightly higher, pH 8.4, and a narrower pH activity range (5.0–9.4). These pH optima are higher than those for other reported β-galactosidases but are well within the range (pH 4.5–9.0) for most of these enzymes (Maksimainen et al., 2012). Michaelis–Menten kinetics were performed at these optimal pHs to determine the kinetic parameters using the *p*NP-β-Gal substrate (Figure 6 and Table 3). For reference, these same assays were also performed using *p*NP-α-Gal at the pH optima, but activity was once again not observed with this substrate (data not shown), consistent with that seen for other β-galactosidases (Maksimainen et al., 2012).

**TABLE 3:** Michaelis–Menten kinetic values calculated for BT2857 and BT3158 *E. coli* LacZ and PvGal-ase.

**Protein**	***V*_max_ (M/min)**	***K*_M_ (M)**	***k*_cat_ (s**^–^**^1^)**	***k*_cat_/*K*_M_ (M**^–^**^1^s**^–^**^1^)**
BT2857	2.32 × 10^–7^ (±7.90 × 10^–9^)	1.3 × 10^–4^ (±2.3 × 10^–5^)	0.046	354
BT2857N	2.29 × 10^–7^ (±1.41 × 10^–8^)	7.0 × 10^–5^ (± 3.0 × 10^–5^)	0.046	657
BT2857C	5.31 × 10^–8^ (±1.63 × 10^–9^)	4.7 × 10^–5^ (±1.0 × 10^–5^)	0.011	234
BT3158	6.06 × 10^–6^ (±1.73 × 10^–7^)	5.2 × 10^–5^ (± 7.5 × 10^–6^)	1.21	23269
*E. coli* LacZ	–	1.2 × 10^–4^	0.0016	13.3
*Schizosaccharomyces pombe* ORF1119 PvGal-ase	–	3.3 × 10^–3^	0.2	60

The catalytic efficiencies (*k*_cat_/*K*_M_) of BT3158 and BT2857 are 2.3 × 10^4^ and 3.5 × 10^2^ M^–1^ s^–1^, respectively. While the value for BT2857 is low compared to other β-galactosidases, BT3158 is within the same range as catalytic efficiencies reported for two *Klebsiella oxytoca* β-galactosidases (β-Gal I and β-Gal II with *k*_cat_/*K*_M_ values of 3.9 × 10^4^ and 7.4 × 10^3^ M^–1^ s^–1^, respectively) using a comparable *o*NP-Gal substrate (Huang et al., 2018). BT2857 and BT3158 kinetics was performed at 20°C whereas the analysis for *K. oxytoca* was conducted at 40°C. However, BT2857 and BT3158 kinetic values are at least an order of magnitude lower than two β-galactosidases from *Bifidobacterium breve* (β-Gal I and β-Gal II with *k*_cat_/*K*_M_ values of 7.2 × 10^5^ and 5.4 × 10^5^ M^–1^ s^–1^, respectively, with an *o*NP-Gal substrate) (Arreola et al., 2014). While these kinetic comparisons are useful, it should be kept in mind that the SusE-like proteins are being assayed outside of their biological context and lacking other protein partners from the SusE-like operon. Moreover, the fact that any activity is observed is a first for this family and of importance to delineating these enzymes further in regard to their carbohydrate utilization patterns and as a means to understanding the function of the overall uncharacterized SusE-like PULs to which they belong.

Following the kinetic analysis with *p*NP-β-Gal, expansion of the substrate was performed. Of the several complex carbohydrates tested as substrates for BT2857, only α-(1,4)-lactose was detected to be degraded by the enzyme. Chemical analysis by the supplier (Millipore Sigma) suggested that β-(1,4)-lactose may make up as much as 4% by mass, which could form the actual substrate for which we observed degradation. BT3158 and the BT2857N truncation showed degradative capacity against α-(1,4)-lactose, while BT2857C did not. The catalytic domain of BT2857 can thus be inferred to exist in the N-terminal portion of SusE-like proteins and not in the DUF5000 domain. Should DUF4959 only serve as a membrane-linker domain with no capacity for binding, as in the analogous E_a_ domain of the prototypical SusE, BT3700, the function must then be associated with the DUF5126; thereby making it a first for this domain grouping.

**FIGURE 7:**
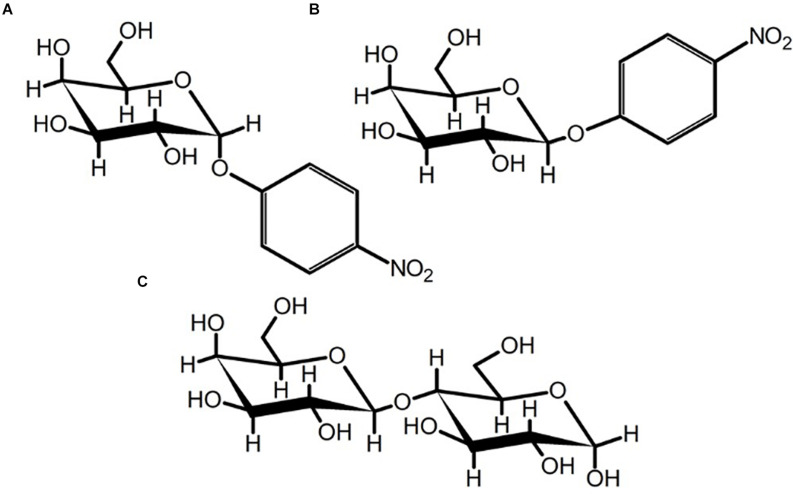
Chair-conformations of **(A)** *p*NP-a-D-galactopyranoside, **(B)** *p*NP-β-D-galactopyranoside, and **(C)** α-(1,4)-lactose. The flexibility of the glycosidic bond in α-(1,4)-lactose may permit accommodation by the SusE-like proteins characterized herein or could be the result of background β-(1,4)-lactose contamination in the sample.

**FIGURE 8:**
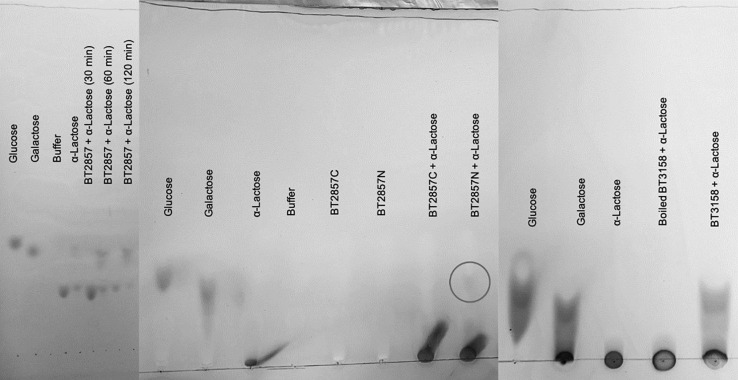
Thin Layer Chromatography analysis of alpha lactose degradation by BT2857 and BT3158. Degradation of α-(1,4)-lactose was observed by BT2857 (full length), BT2857N, and BT3158 by the appearance of a spot further from the origin compared to undigested α-(1,4)-lactose (circled for BT2857N in the middle panel). Figure compiles three separate TLC analyses.

The digestion products of the SusE-like proteins (Figure 7) were then analyzed by TLC to determine their identity by comparison to *R*_f_ values of monosaccharide references in the controls (Figure 8). Clear resolution between product spots was only observed for BT2857, but the migration of spots in all the enzyme samples followed a similar pattern with identifiable spots for a-lactose, galactose and a third compound that had an *R*_f_ value different than glucose. The third compound is less polar than glucose and suggests that the SusE-like proteins may not be typical β-galactosidases operating via hydrolysis (Ardèvol and Rovira, 2015). Instead, the SusE-like proteins appear to have an alternate mechanism (e.g., lyase, dehydratase, etc.) that results in an altered glucose product (such as anhydroglucose, although we are reluctant to suggest a mechanism until the product is characterized by NMR and Mass Spectrometry) that may also account for the kinetic discrepancies we noted.

In seeking to assign putative catalytic residues to the SusE-like family, we performed sequence alignments with the SusE-like proteins we have identified (Supplementary Figure 1). Our analyses were largely limited to these homologs since the prototypical β-galactosidases belong to the GH-A superfamily of glycoside hydrolases that adopt an (α/β)_8_ barrel that is substantially different from the β-stranded domains noted for DUF4959 and DUF5126 of the SusE-like proteins. However, based on our limited dataset, the following residues were highlighted in BT3158 to be homologous, as we had better structural coverage of this protein: C21, D62, D64, D204, and E222. Site-directed mutagenesis of these residues could suggest whether any of these were involved catalytically. Notably, while not sequence conserved, the single Ramachandran outlier in BT2857 is D304. D304 is structurally oriented close to a channel occupied by D256, H265, and Y241. In this channel there are three conserved crystallographic ethylene glycol molecules that are present in both molecules in the asymmetric unit and could suggest the position of carbohydrates for modeling purposes. This region is equivalent to the position for carbohydrate accommodation by CBM family 32. In BT3158 an ethylene glycol and H265 are also structurally conserved. However, this is where the similarities at this region stop in that in BT3158 A304 is in a structurally equivalent position to D304, and none of the other residues between these proteins are conserved in this region suggesting sequence and structural divergence that could also reflect functional variance.

A degradative potential for SusE-like proteins introduces a new paradigm for these proteins as being capable of degrading oligosaccharides, which is in contrast to all previously characterized proteins belonging to this family that have only been shown to bind carbohydrates (Foley et al., 2016; Cartmell et al., 2017). Additionally, these results suggest that one of the two N-terminal DUFs in SusE-like proteins is primarily responsible for this activity, although we are only able to speculate as to the putative catalytic residues. Based on the results found in the literature regarding DUF4959 serving solely as a membrane-linker, as is the case with prototypical SusE proteins (Cameron et al., 2012), this domain is unlikely to be the catalytic module. Thus, we propose that the active domain is the heretofore uncharacterized DUF5126 and propose that the conserved residues of this domain form the catalytic site.

## Conclusion

While the prototypical SusE, BT3700, has been well characterized in the context of the Sus (Shipman et al., 2000; Bateman, 2019), only one of the over one hundred identified SusE-like proteins have been structurally and functionally characterized thus far (Cartmell et al., 2017). In this work we have extended this knowledge by including structural and preliminary functional information for the SusE-like BT2857 and BT3158. We have shown that despite the apparent similarities these proteins share with the prototypical SusE, SusE-like proteins can have significant deviation in both loop regions and secondary structure components of the C-terminal domain that overlaps with DUF5000 (as noted for both BT2857 and BT3158), as well as the DUF5126 in BT3158 when compared to the E_b_ domain of BT3700. Structural deviation was also observed at the region of the E_c_ domain equivalent to the carbohydrate accommodation location of CBM32. As previously seen in the model SusE crystal structure (Cameron et al., 2012), the N-terminal DUF4959 domain in BT3158, representing roughly a quarter of the protein, yielded insufficient data for continuous modeling. The functional work that paralleled these structural studies demonstrated for the first time that these SusE-like proteins, BT2857 and BT3158, are both active against the substrates *p*NP-β-Gal and α-lactose. An N-terminal construct of BT2857, comprised of the DUF4959 and DUF5126 domains, had comparable activity to the full-length BT2857 using *p*NP-β-Gal as a substrate. These functional results, taken together, suggest that the DUF4959/5126 region is capable of activity against carbohydrates containing a terminal C1-bonded β-D-galactose and that certain divergent SusE-like proteins are capable of catalytic activity in addition to the typical carbohydrate-binding function found in both the model SusE and previously characterized SusE-like proteins. Further work to determine the catalytically active residues within this region is ongoing, but we have identified D204 and E222 as potential nucleophile candidates in the DUF5126 (E_b_) domain and D304 in the DUF5000 (E_c_) domain based on sequence conservation and proximity to other conserved residues that may constitute an active site in the crystal structures.

## Data Availability Statement

The datasets presented in this study can be found under the accession codes 7M1A and 7M1B in online respositories. The names of the repository/repositories and accession number(s) can be found below: https://www.rcsb.org/.

## Author Contributions

JS, MS, AB, and MN conceived the work. AB performed initial bioinformatics analysis and construction of genome-derived recombinant plasmids. JS and MN produced protein and conducted crystallization and functional assays. MS and JW conducted diffraction analysis model building and refinement. JS processed SAXS data and model building. JS, JW, and MS wrote the manuscript. All authors contributed to the article and approved the submitted version.

## Conflict of Interest

The authors declare that the research was conducted in the absence of any commercial or financial relationships that could be construed as a potential conflict of interest.

## Footnotes

1 http://www.bioisis.net/tutorials/9

2 https://bl1231.als.lbl.gov/ran

3 http://saxs.ifsc.usp.br/

4 http://raptorx.uchicago.edu/StructPredV2/predict/

## Supplementary Material

Supplementary Figure 1 Sequence alignment of six SusE-like proteins from *B. thetaiotaomicron* ATCC 29148. Residues highlighted are conserved (^∗^), highly similarity (:) or have low similarity (.) and positional number is provided below the sequences is for BT3158. A conserved cysteine (C21, yellow) is the candidate for lipidation and attachment at the outer membrane. Side chain residues that are contributed to a calcium binding site observed in BT2857 and CBM32 are highlighted pink. Putative catalytic residues D62/D64 and E222 are present in the N-terminal or E_a_ domain DUF4959. A structurally conserved histidine residue (H265) present in the carbohydrate accommodation site of CBM32 is also conserved in BT2857 and BT3158.

Supplementary Table 1 PCR primers used for cloning BT2857 and BT3158 from genomic DNA.
