# Supplementary figures and images for: Analysis of Two SusE-Like Enzymes From Bacteroides thetaiotaomicron Reveals a Potential Degradative Capacity for This Protein Family
Source: Front Microbiol. 2021 Jun 4;12:645765. doi: 10.3389/fmicb.2021.645765 (PMC8211771; doi:10.3389/fmicb.2021.645765)

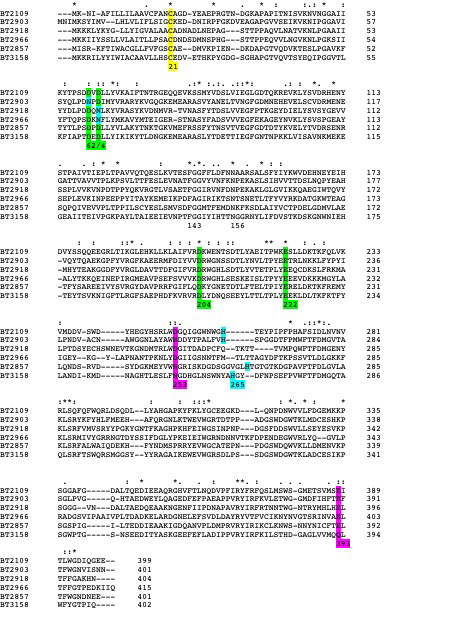

Supplement: Supplementary Figure 1 — Sequence alignment of six SusE-like proteins from B. thetaiotaomicron ATCC 29148. Residues highlighted are conserved (∗), highly similarity (:) or have low similarity (.) and positional number is provided below the sequences is for BT3158. A conserved cysteine (C21, yellow) is the candidate for lipidation and attachment at the outer membrane. Side chain residues that are contributed to a calcium binding site observed in BT2857 and CBM32 are highlighted pink. Putative catalytic residues D62/D64 and E222 are present in the N-terminal or Ea domain DUF4959. A structurally conserved histidine residue (H265) present in the carbohydrate accommodation site of CBM32 is also conserved in BT2857 and BT3158. [file Image_1.JPEG]
