# Supplementary material for: Analysis of Two SusE-Like Enzymes From Bacteroides thetaiotaomicron Reveals a Potential Degradative Capacity for This Protein Family
Source: Front Microbiol. 2021 Jun 4;12:645765. doi: 10.3389/fmicb.2021.645765 (PMC8211771; doi:10.3389/fmicb.2021.645765)
Supplement: Supplementary Table 1 — PCR primers used for cloning BT2857 and BT3158 from genomic DNA. [file Table_1.DOCX]

# Supplementary Material

**Supplemental Table S1. PCR primers used for cloning BT2857 and BT3158 from genomic DNA.**

| **Primer** | **Nucleotide Sequence (5’ – 3’)** |
| --- | --- |
| BT2857Forward | CAGGGACCCGGTGCAGAAGATATGGTAAAGCCCATTG |
| BT2857Reverse | CGAGGAGAAGCCCGGTTATTCTTCGTTATCATTCCCCCAGAAG |
| BT3158Forward | CAGGGACCCGGTGAAGATGTAGAAACTCATAAACCTTATG |
| BT3158Rverese | CGAGGAGAAGCCCGGTTATTGTATCGGGGTTCCGTAGAACC |

**Supplemental Figure 1. Sequence alignment of six SusE-like proteins from *B. thetaiotaomicron* ATCC 29148**. Residues highlighted are conserved (*), highly similarity (:) or have low similarity(.) and positional number is provided below the sequences is for BT3158. A conserved cysteine (C21, yellow) is the candidate for lipidation and attachment at the outer membrane. Side chain residues that are contributed to a calcium binding site observed in BT2857 and CBM32 are highlighted pink. Putative catalytic residues D62/D64 and E222 are present in the N-terminal or E_a_ domain DUF4959. A structurally conserved histidine residue (H265) present in the carbohydrate accommodation site of CBM32 is also conserved in BT2857 and BT3158.
